# Exploration of various administration routes for the nitric oxide donating organic nitrite, nitrosooxypropanol, in a porcine model

**DOI:** 10.1080/10717544.2026.2752663

**Published:** 2026-11-04

**Authors:** Anna Stene Hallerbäck, Måns Edström, Per Agvald, Kristofer F. Nilsson

**Affiliations:** a School of Medical Sciences, Örebro University, Örebro, Sweden; b Department of Cardiothoracic and Vascular Surgery, Faculty of Medicine and Health, Örebro University, Örebro, Sweden; c Department of Anesthesiology and Intensive Care, Faculty of Medicine and Health, Örebro University, Örebro, Sweden; d Department of Physiology and Pharmacology, Section of Pharmacology, Karolinska Institutet, Stockholm, Sweden; e Attgeno AB, Stockholm, Sweden

**Keywords:** Nitric oxide, nitric oxide donor, organic nitrite, administration routes, pulmonary circulation, pulmonary hypertension

## Abstract

Organic nitrites are restricted to intravenous or inhaled administration. The aim was to explore novel administration routes for an organic nitrite, previously demonstrated to have pulmonary selectivity, in normal and elevated pulmonary vascular resistance. Hemodynamic and respiratory variables, including fraction of exhaled nitric oxide (FeNO), were measured in 13 anesthetized pigs. In naïve conditions, nitrosooxypropanol (PDNO) was given subcutaneously (100–1600 nmol/kg/min; 5 min/dose, 30 min interval), intramuscularly (50–400 nmol/kg/min; 5 min/dose, 30 min interval), intravenously (5–80 nmol/kg/min; 30 min infusion), intranasally (500–2000 nmol/kg boli), and sublingually (80–16,240 nmol/kg boli). In pulmonary hypertension (intravenous infusion of U46619), PDNO was given intravenously (5–45 nmol/kg/min; 15–20 mins infusion/dose) and subcutaneously (200–1800 nmol/kg/min; 5 min/dose at 20 min interval). Recovery was calculated using FeNO. PDNO, given by all administration routes, dose-dependently decreased systemic blood pressure and increased FeNO. Subcutaneous and intramuscular administration induced slow-onset (9 min to maximum effect) and long-lasting effects (~30 min). Intravenous administration induced rapid effects (1–2 min to steady state) and prompt decline after infusion cessation. The proportion of PDNO reaching exhaled gas (intravenous administration as reference; FeNO-based recovery) was 17% (intramuscularly) and 9% (subcutaneously). In pulmonary hypertension, intravenous and subcutaneous administration decreased the pulmonary/systemic vascular resistance-ratio. In this exploratory proof-of-concept study evaluating novel administration routes for organic nitrites, PDNO demonstrated measurable biological effects and acceptable FeNO-based recovery following intramuscular and subcutaneous administration. Furthermore, intranasal and tentatively sublingual administration showed biological effects. Future work on formulation and investigations into relevant disease models is warranted.

## Introduction

Organic nitrites have been proposed as therapeutic agents in patients with pulmonary hypertension (Moya et al. 2002; Romanelli et al. 2004; Auten et al. 2007). The nitric oxide (NO) pathway is widely recognized as a significant mediator in the pathogenesis of pulmonary hypertension, but there are few drugs affecting the NO pathway currently on the market (i.e. phospodisesterase-5 inhibitors, soluble guanylate cyclase stimulators, and inhaled nitric oxide) (Frostell et al. 1991, 1993; Stasch et al. 2001; Galie et al. 2005; Rubin et al. 2011). One reason is that many intravenously (i.v.) administered drugs acting via this pathway are not selective for the pulmonary circulation, and may, therefore, trigger unwanted systemic hypotension (McLean et al. 1990; Cockrill et al. 2001; Miller and Megson 2007). On the other hand, inhaled NO does not affect systemic blood pressure, instead reaching only ventilated regions of the lung, and becoming inactivated when exposed to hemoglobin (Lambermont et al. 1999; Guarin et al. 2001).

It is well known that volatile organic nitrites can be inhaled. Organic nitrites, such as ethyl nitrite, have been proposed as therapeutic agents for inhalation in newborns with persistent pulmonary hypertension (Moya et al. 2002; Auten et al. 2007). Furthermore, amyl nitrite has been shown to decrease pulmonary pressure and vascular resistance in a pig model of thromboxane-induced pulmonary hypertension, and is used in treating cyanide intoxication and also recreationally as an inhalant drug (Romanelli et al. 2004).

When administered i.v., the novel organic nitrite nitrosooxypropanol (PDNO) has been shown to effectively decrease pulmonary arterial pressures and lower pulmonary vascular resistance (PVR) in animal models of acute pulmonary hypertension (Nilsson et al. 2011, 2018; Stene Hurtsen et al. 2020). Nitrosooxypropanol consists of organic mono-nitrites synthesized with 1,2-propanediol as a carrier. Nitrosooxypropanol has a presumed ultra-short half-life due to its fast decomposition in the blood. When administered i.v. at low doses, it has a vasodilatory effect that is primarily confined to the lungs due to the majority of bound NO being released during its first passage through the pulmonary circulation. This local pulmonary vasodilatory effect, along with the absence of severe systemic effects, has been investigated across various animal models representing different etiologies of pulmonary hypertension (Nilsson et al. 2018; Nilsson and Gustafsson 2019; Stene Hurtsen et al. 2020; Nilsson et al. 2020). Intravenous administration of PDNO has been investigated in healthy volunteers (NCT04891354) and in patients undergoing cardiac surgery with postoperative PH (NCT05699486 and EudraCT No. 2021-005032-30).

Apart from i.v. delivery and inhalation, administration routes for organic nitrites have not been thoroughly investigated. Potential alternative routes may be subcutaneous (s.c.) or intramuscular (i.m.) delivery, which would facilitate easier administration in certain chronic conditions. For example, in chronic pulmonary arterial hypertension (PAH), prostacyclin can be administered s.c. (Simonneau et al. 2002). Inorganic nitrites have been proposed for the treatment of PAH, delivered in preclinical studies through s.c., i.m., intraperitoneal, and nebulized routes. While they demonstrate some degree of efficacy, the overall therapeutic effect seems to be relatively modest (Vick and Von Bredow 1996; Zuckerbraun et al. 2010; Pankey et al. 2012; Honavar et al. 2017; Kawaguchi et al. 2017; Jankov et al. 2018; Munzel and Daiber 2018). Organic nitrates, including nitroglycerin, have been studied and formulated for various routes, such as oral, sublingual, buccal, and transdermal administration. However, the development of tolerance has posed challenges in clinical long-term use (Parker and Parker 1998; Agvald et al. 1999). In contrast to organic nitrates, PDNO use does not appear to be restricted by tolerance development (Nilsson et al. 2018; Stene Hurtsen et al. 2020). While previous studies have primarily investigated i.v. or inhalational delivery of organic nitrites, other administration routes, including s.c., i.m., intranasal, and sublingual, have not, to the best of our knowledge, been shown for organic nitrites. If organic nitrites, such as PDNO, can be administered via routes other than i.v. and inhalation, patient-administered treatment for chronic conditions, e.g. PAH, may be feasible.

The objective of this study was to investigate, within a comprehensive experimental framework in a large-animal model, the fundamental pharmacological properties of PDNO administration via different routes in anesthetized pigs, under conditions of both normal and elevated PVR, as proof of concept for the development of patient-controlled administration methods for chronic use.

## Material and methods

Ethical approval was received from Linköping’s Regional Animal Ethics Committee (Linköping, Sweden; approval number 953, reference number 5.2.18-637/16) and conducted at a licensed animal research facility (license number 35-4415/03). Thirteen male and female pigs (a crossbreed between Swedish country breed, Hampshire, and Yorkshire; 3–4 months old; 24–26 kg) were used. On each experimental day, the animals were transported to the laboratory following i.m. premedication with azaperone (6–7 mL, 40 mg/mL; Stresnil Elanco, Herlev, Denmark) administered prior to the 30-min transport. Upon arrival, anesthesia was induced i.m. with zolazepam/tiletamine (3 mg/kg of each substance, Zoletile Forte; Virbac, Kolding, Denmark) in combination with azaperone (4 mg/kg), and atropine (1.5 mg, i.m.; Mylan, Stockholm, Sweden) was given. Following orotracheal intubation with a 6 mm endotracheal tube (Covidien, Tullamore, Ireland), the animals were connected to a ventilator (PV 501, Breas Medical AB, Sweden) and maintained under continuous i.v. infusions of propofol (5–15 mg/kg/h, Fresenius Kabi, Uppsala, Sweden), remifentanil (30–50 μg/kg/h, Meda, Solna, Sweden), 5% glucose (0.5 mL/kg/h, Fresenius Kabi), and Ringer’s acetate (10 mL/kg/h, Fresenius Kabi). Infusions were delivered using calibrated infusion pumps (Alaris GP; Cardinal Health Care, Rolle, Switzerland). Mechanical ventilation was set with a positive end-expiratory pressure (PEEP) of 5 cm H₂O. If signs of inadequate anesthesia or analgesia could not be corrected, the experiment was terminated, and the animal washumanely euthanized. The ARRIVE guidelines were followed as closely as practicable (Percie du Sert et al. 2020).

Arterial access for blood sampling (methemoglobin [MetHb], hemoglobin [Hb], and arterial blood gases) and continuous monitoring of mean arterial pressure (MAP) and heart rate (HR) was established via a 5 Fr catheter (Avanti, Cordis, Cashel, Ireland) inserted into the right common carotid artery using the Seldinger technique. Arterial blood gases were measured using Gem 4000 (Instrumental Laboratory, Lexington, MA, USA). A pulmonary arterial catheter (7.5 Fr, Swan-Ganz CCOmbo, Edwards Lifesciences, Irvine, CA, USA) was introduced through a 9 Fr (Avanti, Cordis) introducer sheath in the right external jugular vein to enable measurement of pulmonary arterial pressure, central venous pressure (CVP), continuous cardiac output (CO; thermodilution technique), and body temperature. Pulmonary capillary wedge pressure (PCWP) was obtained by intermittent balloon inflation. Additional venous access for fluid and drug administration was secured via a central venous catheter (Avanti, Cordis) placed in the left external jugular vein. Hemodynamic variables and ventilatory parameters including tidal volume (TV), minute ventilation (MV), respiratory rate (RF), inspired oxygen fraction (FiO₂), end-tidal oxygen (ETO₂), end-tidal carbon dioxide (ETCO₂), positive end-expiratory pressure (PEEP), and peak inspiratory pressure (PIP) were continuously monitored (ICU monitor, AS/3, Datex, Helsinki, Finland) and digitally recorded using the MP150/Acknowledge 3.9.1 system (BIOPAC systems; Goleta, CA, USA). The fraction of NO, was measured and recorded continuously via an endotracheal tube using chemiluminescence (CLD 77, ECO PHYSICS, Switzerland). The fraction of exhaled NO (FeNO) was extracted from the fraction of NO using the peak maximum detection tool in the Acknowledge software. The urinary bladder was catheterized (Foley, Bard Limited, Crawley, UK) via a midline laparotomy (3–4 cm incision). Following completion of instrumentation, 5000 IU heparin *n* (LEO Pharma, Malmö, Sweden) was administered i.v. Animals were covered with warming blankets to maintain normothermia. A stabilization period of 1 h without intervention was allowed to achieve steady baseline conditions before initiation of the experimental protocol. Upon completion of the experimental procedures, cardiac arrest was induced under deep anesthesia by i.v. administration of potassium chloride (Braun, Danderyd, Sweden). Circulatory arrest was confirmed by electrocardiography, invasive blood pressure monitoring, and absence of ETCO₂.

### Experimental protocol

One hour of intervention-free time followed the surgical preparation. The experimental protocol was set to maximize the number of interventions in each animal, considering washout and stabilization periods. After collecting baseline data, i.m., s.c., and intranasal routes for the administration of PDNO (RISE, Södertälje, Sweden) were investigated in the same animal in non-randomized order, with a washout period of at least 1 h between each administration. Intravenous administrations in naïve (pulmonary normotensive) animals, and i.v. and s.c. administrations in pulmonary hypertensive animals, were investigated in the same animals with a washout period of at least 1 h between administrations. The duration of the wash-out periods was set to allow all animals to return to baseline hemodynamic status and FeNO values. No PDNO administration was started before baseline values were reached. No blinding was performed due to practical reasons. Sublingual administration was performed in a separate animal. The same composition, formulated for i.v. use, was used for all routes of administration.

#### Experiments at normal pulmonary vascular resistance

The doses chosen for the experiments were set after a pilot experiment to achieve a similar response on FeNO when PDNO was administered i.v., i.m., and s.c. to enable pharmacological calculations utilizing FeNO (see below). Intravenous infusions of PDNO into a carrier flow of a solution of sodium bicarbonate (1.4%; pH approximately 8; infusion rate 9 times the PDNO infusion rate) in increasing doses (5, 10, 20, 40, and 80 nmol/kg/min, *n* = 6 were performed for 30 min at each dose. Subcutaneous (in the neck) and i.m. (gluteal muscles) infusions in increasing doses (s.c.: 100, 200, 400, 800, and 1600 nmol/kg/min, *n* = 6; i.m: 50, 100, 200 and 400 nmol/kg/min, *n* = 4–6) were delivered for 5 min followed by 25 min observation time per dose. Intranasal bolus applications of PDNO in two doses (500 and 2000 nmol/kg, *n* = 6) were administered into one nostril, with 15 min observation time per dose. Sublingual administration was achieved using a PDNO-packed sponge (2 mL) under the tongue in five doses (80, 800, 2400, 8000 and 16240 nmol/kg, *n* = 1), with 10 min observation time per dose. Arterial blood gases were sampled at baseline, and at 10, 15, and 25 min after the start of infusion in the s.c., i.m., and i.v. experiments. Primary outcome measures were mean pulmonary arterial pressure (MPAP), PVR, and FeNO.

#### Experiments on increased pulmonary vascular resistance

Mean pulmonary arterial pressure was increased to approximately 35  mmHg by a continuous i.v. infusion of U46619 (a synthetic and stable analog of thromboxane A_2_, Cayman Chemical, MI, USA). Intravenous and s.c. infusions of PDNO in increasing doses (i.v.: 5, 15, and 45 nmol/kg/min, *n* = 6 for 15 min at each dose; s.c.: 200, 600, and 1800 nmol/kg/min, *n* = 7 were delivered for 5 min, followed by 10 min observation time per dose, in a non-randomized manner. Arterial blood gases were sampled at baseline, after stabilization of U46619 infusion, and at 13 min after start of PDNO infusion (i.v. and s.c.). Primary outcome measures were MPAP, PVR, and FeNO.

#### Statistics

An a priori power calculation was not feasible due to the exploratory nature of the study design and the lack of preliminary or published data. Descriptive data are presented as mean with standard error of the mean (SEM), if not otherwise stated. A small proportion of data points were missing at random. Shapiro-Wilk’s test was used to evaluate normality. For time- and dose-dependent effects on FeNO and hemodynamic variables, linear mixed models were used to compare baseline to maximum effect (9 min after start of infusion) with Bonferroni correction for multiple comparisons using SPSS (version 27, IBM Corp., Armonk, NY, USA). Fixed effects were route of administration and dose, with subject as a random effect. For comparisons between administration routes in naïve experiments of i.v., s.c., and i.m. administration, a linear mixed model was fitted (*lme* from *nlme* (Pinheiro 2025)) using R 4.4.3. (R Core Team 2026). For approximation of FeNO-based PDNO recovery, the response variable, cumulative fraction FeNO (FeNO_C_ [ppb]), underwent a log transformation to address heteroscedasticity, while doses were log_10_-transformed. Log[FeNO_C_] was modeled as dependent on route of administration (categorical), log_10_[dose] (continuous), and the interaction of route of administration and log_10_[dose] (fixed effects). Subject was considered as a random effect. To facilitate dosage comparisons across routes, i.v. FeNO_C_ was estimated at 500, 1000, and 2000 nmol/kg (2.7, 3.0, and 3.3 on log_10_-scale) using the fitted model. Coefficients from the fitted models were extracted and compared using *emtrends* and *pairs* (*emmeans* (Lenth 2025)). For estimation of FeNO-based half-life of s.c. and i.m. infusions, non-linear mixed models were fitted according to the function $y\left(t\right)=e^{-\mathrm{kt}}$ (using *nlme* from *nlme* (Pinheiro 2025)). Peak FeNO normalized to [0.0 … 1.0] was used, and the subject was treated as a random effect. CI_95%_ were estimated around the *k* coefficients using bootstrapping. Half-lives were then calculated using $t_{1/2}=\;\log2/k$ . No statistical analyses were performed on the sublingual experiments. Statistical significance was considered at *P* < 0.05.

## Results

### Effects of PDNO in naïve animals

In normal PVR, i.v., s.c., and i.m., the administration of PDNO caused dose-dependent increases in FeNO and reductions in MAP (Figures 1 and 2). Time to maximal effect on FeNO was 9.4 ± 1.0 and 9.4 ± 0.9  min during i.m. and s.c. administrations, respectively. The effects were more long-lasting compared to i.v. infusion. Intravenous infusion caused a rebound effect on MAP after halting infusion (Figure 1). Systemic vascular resistance was significantly decreased by PDNO at 40 nmol/kg during i.v. administration and at 400 nmol/kg/min in the s.c. and i.m. administration routes (Table 1). Intramuscular PDNO administration significantly decreased PVR at 400 nmol/kg/min (Table 1). Cumulative exhaled NO (FeNO_C_) levels were significantly higher relative to dose when comparing i.v. with i.m. and s.c. administrations, as well as when comparing i.m. with s.c. administrations (Figure 3). The dose ratio in terms of fraction of FeNO-based recovery for i.v.: i.m.: s.c. administration was approximately 1.0:5.9:11.1 suggesting that approximately 17% and 9% of the PDNO doses administered i.m. and s.c, respectively, were biologically available as cumulative exhaled NO when using i.v. administration as the reference. Estimated half-life of FeNO response following s.c. and i.m. PDNO administration was 20.5 (CI_95%_ 14.8–36.4) min and 6.2 (CI_95%_ 5.1–7.4) min, respectively.

**Table 1.** Hemodynamics and blood gases after infusion of nitrosooxypropanol (PDNO) in normal pulmonary vascular resistance.

	*PDNO*			*HR* *(beats/min)*		*CO* *(L/min)*		*CVP* *(mm Hg)*							*PCWP* *(mm Hg)*		*SVR* *(mm Hg/L/min)*	*PVR (mm Hg/L/min)*	*pH*	*pO*_*2*_ *(kPa)*	*pCO*_*2*_ *(kPa)*	*MetHb* *(%)*
I.v.																						
	Baseline			99 ± 6		4.6 ± 0.4		10 ± 1							12 ± 1		14.8 ± 0.5	1.9 ± 0.1	7.54 ± 0.01	12.2 ± 0.2	4.8 ± 0.2	2.0 ± 0.1
	**5 nmol/kg/min**																					
	10 min			101 ± 6		4.6 ± 0.4		10 ± 0							12 ± 0		14.9 ± 0.9	2.1 ± 0.4	7.54 ± 0.01	11.7 ± 0.3	4.7 ± 0.1	2.0 ± 0.3
	25 min			104 ± 7		4.7 ± 0.3		10 ± 0							12 ± 0		13.6 ± 1.1	1.9 ± 0.3	7.54 ± 0.01	11.7 ± 0.2	4.6 ± 0.1	2.4 ± 0.2
	**10 nmol/kg/min**																					
	10 min			104 ± 6		4.6 ± 0.3		10 ± 0							12 ± 0		14.0 ± 0.5	1.8 ± 0.2	7.55 ± 0.01	11.7 ± 0.2	4.6 ± 0.1	2.0 ± 0.3
	25 min			105 ± 7		4.7 ± 0.3		10 ± 0							12 ± 1		13.2 ± 0.4	1.7 ± 0.2	7.55 ± 0.01	11.7 ± 0.3	4.5 ± 0.1	2.1 ± 0.3
	**20 nmol/kg/min**																					
	10 min			105 ± 7		4.7 ± 0.3		10 ± 1							12 ± 1		12.7 ± 0.5	1.7 ± 0.2	7.56 ± 0.01	11.8 ± 0.4	4.5 ± 0.1	2.0 ± 0.2
	25 min			106 ± 7		4.8 ± 0.3		10 ± 1							12 ± 1		12.6 ± 0.8	1.6 ± 0.2	7.56 ± 0.01	11.5 ± 0.4	4.5 ± 0.1	2.1 ± 0.1
	**40 nmol/kg/min**																					
	10 min			106 ± 7		4.6 ± 0.3		9 ± 0							12 ± 1		11.5 ± 0.6*	1.5 ± 0.2	7.57 ± 0.01	10.9 ± 0.3*	4.5 ± 0.1	1.9 ± 0.3
	25 min			109 ± 7		4.9 ± 0.4		10 ± 0							12 ± 1		11.3 ± 0.4	1.4 ± 0.2	7.57 ± 0.01	11.0 ± 0.4	4.5 ± 0.1	2.2 ± 0.2
	**80 nmol/kg/min**																					
	10 min			108 ± 7		4.7 ± 0.3		10 ± 0							12 ± 0		11.1 ± 0.6*	1.4 ± 0.1	7.57 ± 0.01	10.3 ± 0.5*	4.5 ± 0.1	1.8 ± 0.3
	25 min			114 ± 8		5.3 ± 0.4		10 ± 1							12 ± 0		9.9 ± 0.5	1.3 ± 0.2	7.56 ± 0.01	10.2 ± 0.6	4.5 ± 0.1	2.0 ± 0.2
S.c																						
	Baseline			103 ± 5	5.2 ± 0.3			10 ± 1							11 ± 1		14.7 ± 1.4	1.6 ± 0.2	7.57 ± 0.01	12.0 ± 0.2	4.7 ± 0.1	1.8 ± 0.3
	**100 nmol/kg/min**																					
	10 min			103 ± 5		5.2 ± 0.3		9 ± 0							11 ± 1		13.9 ± 1.3	1.6 ± 0.1	7.57 ± 0.01	11.8 ± 0.1	4.7 ± 0.1	1.6 ± 0.4
	25 min			105 ± 5		4.8 ± 0.5		8 ± 0							10 ± 1		14.1 ± 1.2	1.6 ± 0.1	7.57 ± 0.00	12.1 ± 0.2	4.7 ± 0.1	1.9 ± 0.1
	**200 nmol/kg/min**																					
	10 min			108 ± 5		5.2 ± 0.3		10 ± 1							11 ± 1		13.5 ± 1.1	1.5 ± 0.1	7.58 ± 0.01	11.9 ± 0.3	4.5 ± 0.1	1.8 ± 0.2
	25 min			110 ± 5		4.8 ± 0.3		10 ± 1							11 ± 1		13.4 ± 0.9	1.7 ± 0.1	7.58 ± 0.01	12.0 ± 0.2	4.4 ± 0.1	1.8 ± 0.2
	**400 nmol/kg/min**																					
	10 min			107 ± 5		5.6 ± 0.4		9 ± 1							11 ± 1		10.5 ± 0.6^*^	1.2 ± 0.1	7.59 ± 0.01	11.5 ± 0.3	4.3 ± 0.1	1.8 ± 0.3
	25 min			109 ± 5		5.6 ± 0.3		9 ± 1							11 ± 1		11.9 ± 0.8	1.5 ± 0.1	7.58 ± 0.01	11.9 ± 0.4	4.4 ± 0.1	2.1 ± 0.2
	**800 nmol/kg/min**																					
	10 min			106 ± 5		5.5 ± 0.5		9 ± 1							11 ± 1		10.4 ± 0.8^*^	1.3 ± 0.1	7.58 ± 0.01	10.8 ± 0.2*	4.5 ± 0.1	2.0 ± 0.2
	25 min			110 ± 6		5.5 ± 0.4		10 ± 1							11 ± 1		12.1 ± 0.9	1.5 ± 0.2	7.58 ± 0.01	11.6 ± 0.2	4.5 ± 0.1	1.6 ± 0.2
	**1600 nmol/kg/min**																					
	10 min			105 ± 5		5.1 ± 0.5		9 ± 1							11 ± 1		10.5 ± 1.0^*^	1.3 ± 0.1	7.58 ± 0.01	10.5 ± 0.2*	4.4 ± 0.1	1.8 ± 0.2
	25 min			108 ± 5		5.6 ± 0.4		10 ± 1							11 ± 1		12.0 ± 1.3	1.4 ± 0.1	7.58 ± 0.01	11.1 ± 0.4	4.5 ± 0.1	2.1 ± 0.3
I.m.																						
	Baseline			114 ± 4		5.6 ± 0.2		10 ± 1							11 ± 2		13.8 ± 1.0	2.2 ± 0.2	7.58 ± 0.00	11.0 ± 0.3	4.6 ± 0.1	1.7 ± 0.1
	**50 nmol/kg/min**																					
	10 min			114 ± 5		5.6 ± 0.2		9 ± 1							10 ± 1		13.0 ± 1.4	2.1 ± 0.3	7.57 ± 0.01	10.4 ± 0.1	4.6 ± 0.1	2.0 ± 0.3
	25 min			113 ± 4		5.7 ± 0.5		9 ± 1							10 ± 1		13.1 ± 1.7	2.2 ± 0.3	7.57 ± 0.01	10.8 ± 0.4	4.6 ± 0.0	2.2 ± 0.3
	**100 nmol/kg/min**																					
	10 min			114 ± 3		5.6 ± 0.1		9 ± 1							11 ± 1		12.0 ± 1.1	1.8 ± 0.1	7.58 ± 0.01	11.0 ± 0.3	4.5 ± 0.1	1.7 ± 0.2
	25 min			115 ± 3		5.7 ± 0.2		10 ± 1							11 ± 1		12.8 ± 1.2	2.0 ± 0.2	7.58 ± 0.01	11.2 ± 0.3	4.5 ± 0.1	1.8 ± 0.4
	**200 nmol/kg/min**																					
	10 min			110 ± 2		5.3 ± 0.3		10 ± 1							11 ± 1		13.2 ± 1.1	1.9 ± 0.2	7.58 ± 0.01	10.7 ± 0.3	4.4 ± 0.1	1.8 ± 0.2
	25 min			109 ± 2		5.5 ± 0.3		10 ± 1							11 ± 1		14.0 ± 1.5	2.3 ± 0.1	7.57 ± 0.01	10.8 ± 0.4	4.5 ± 0.2	1.8 ± 0.3
	**400 nmol/kg/min**																					
	10 min			111 ± 5		5.4 ± 0.3		8 ± 0							11 ± 1		10.6 ± 1.1^*^	1.4 ± 0.1^*^	7.58 ± 0.01	9.4 ± 0.3	4.5 ± 0.1	2.1 ± 0.2
	25 min			107 ± 4		5.5 ± 0.4		10 ± 1							12 ± 1		14.1 ± 1.5	2.1 ± 0.2	7.58 ± 0.01	10.6 ± 0.4	4.5 ± 0.2	2.0 ± 0.2

^*^ Represents statistical significance (*P* < 0.05) from baseline to maximum effect (9 min after start of infusion).

Notes: Heart rate (HR), cardiac output (CO), central venous pressure (CVP), pulmonary capillary wedge pressure (PCWP), systemic vascular resistance (SVR), pulmonary vascular resistance (PVR), pH, partial pressure of oxygen (pO_2_), partial pressure of carbon dioxide (CO_2_), and methemoglobin levels (MetHb) during intravenous (i.v., 5–80 nmol/kg/min for 30 min each, *n* = 6), subcutaneous (s.c., 100–1600 nmol/kg/min for 5 min each, *n* = 6) and intramuscular (i.m., 50–400 nmol/kg/min for 5 min each, *n* = 4–6) infusion of nitrosooxypropanol (PDNO) in anesthetized pigs. Data are expressed as means with standard errors of the means (SEM).

**Figure 1.**
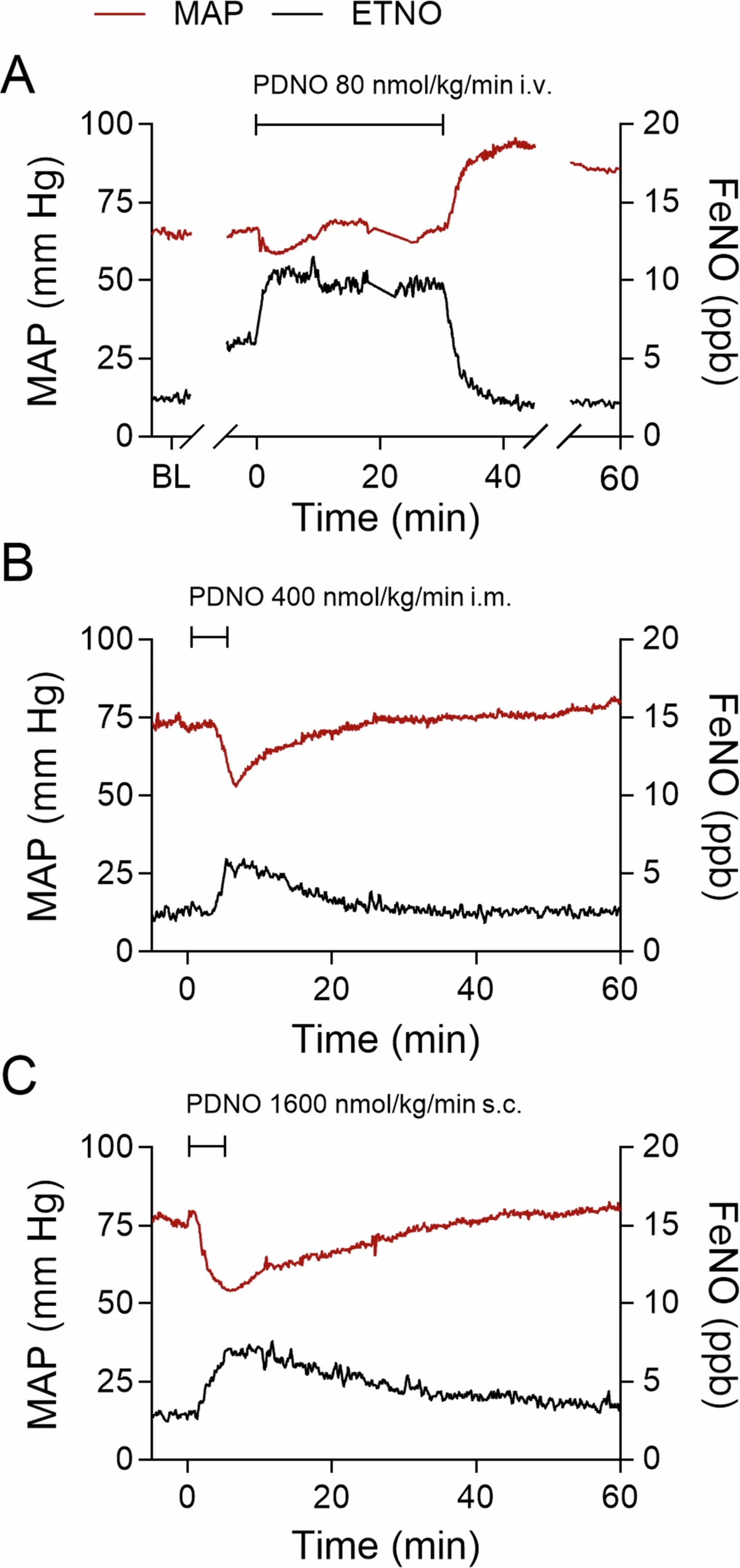
Original recordings (*n* = 1) of mean arterial blood pressure (MAP, red line) and fraction of exhaled nitric oxide (FeNO, black line) during infusions of nitrosooxypropanol (PDNO) intravenously (i.v.; 80 nmol/kg/min, 30 min infusion, panel A), intramuscularly (i.m.; 400 nmol/kg/min, 5 min infusion, panel B) and subcutaneously (s.c.; 1600 nmol/kg/min, 5 min infusion, panel C) in an anesthetized pig.

**Figure 2.**
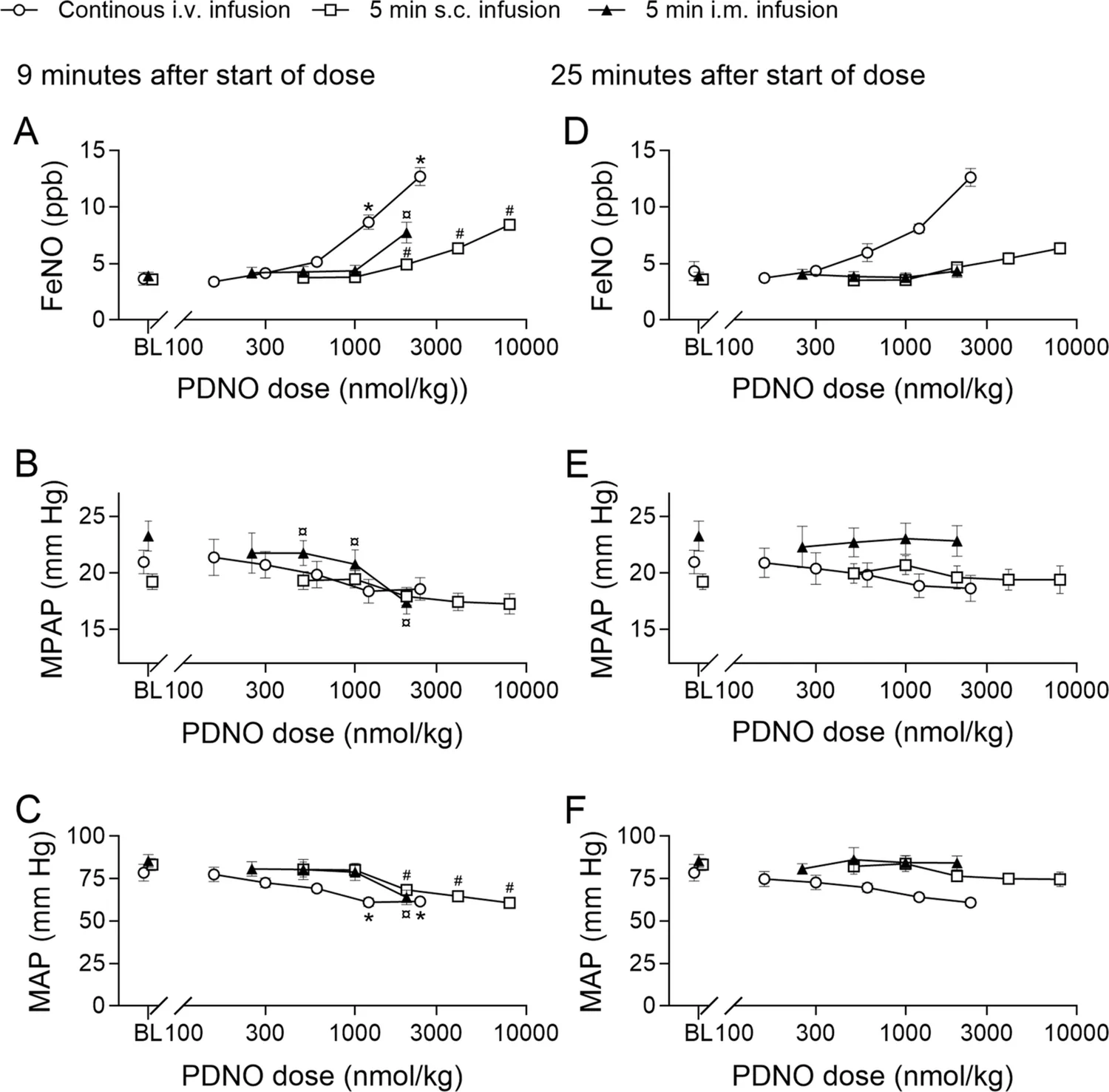
Effects of nitrosooxypropanol (PDNO) on fraction of exhaled nitric oxide (FeNO, panel A and D), mean pulmonary arterial pressure (MPAP, panel B and E), and mean arterial pressure (MAP, panel C and F) at 9 (panel A, B, C), and 25 (panel D, E, F) min, administered intravenously (i.v.; 5–80 nmol/kg/min for 30  min each, *n* = 6), subcutaneously (s.c.; 100–1600 nmol/kg/min for 5  min each, *n* = 6) and intramuscularly (i.m.; 50–400 nmol/kg/min for 5  min each, *n* = 4–6) in anesthetized naïve pigs. Data are expressed as means with standard error of the mean. *, ^¤^ , and ^#^ represent statistical significance (*P* < 0.05) from baseline (BL) at 9 min (panel A) for i.v., i.m., and s.c. infusions, respectively.

**Figure 3.**
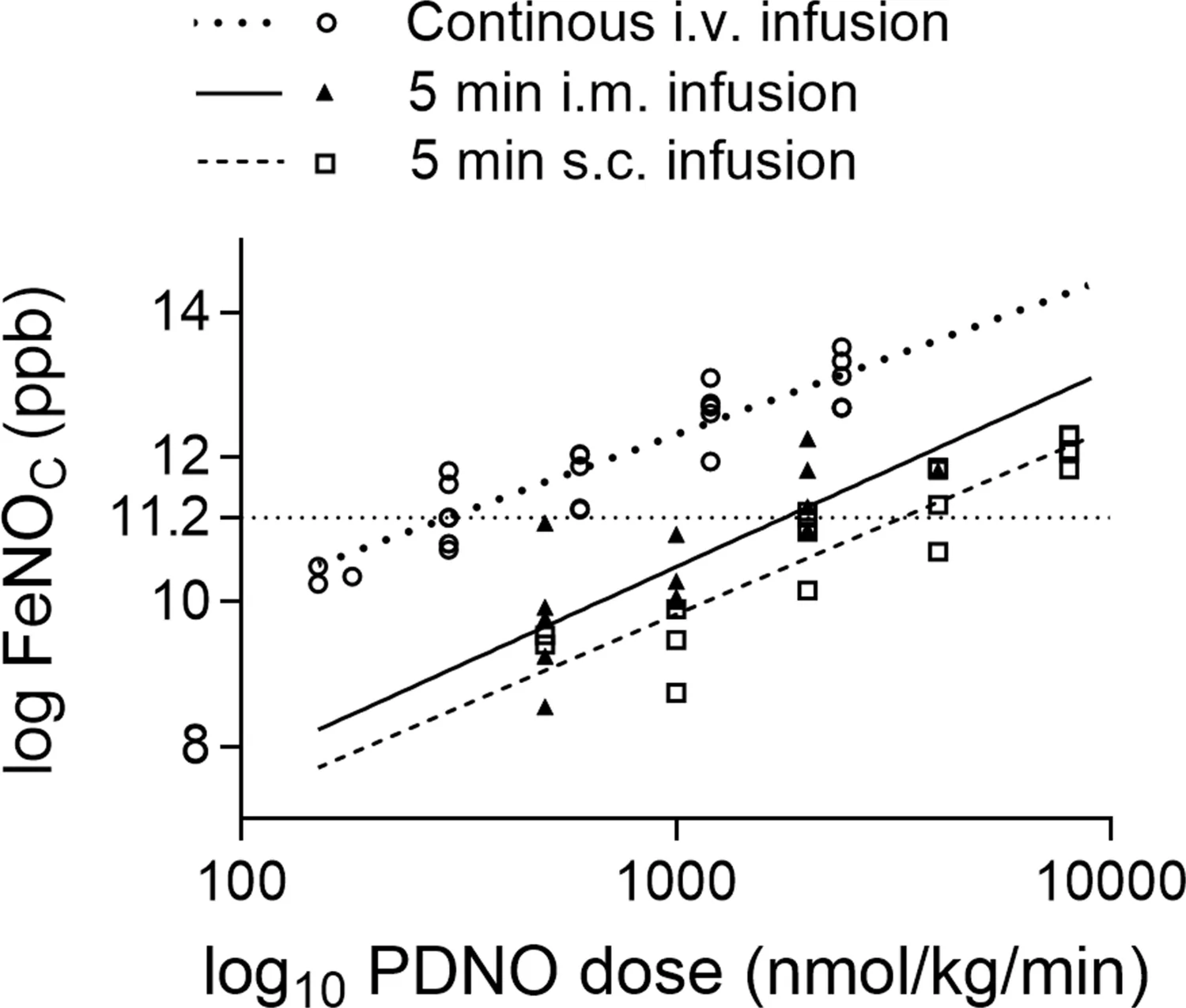
Logarithmized cumulative exhaled NO (log FeNO_C_) measured over a 30-min period after administration of nitrosooxypropanol (PDNO) intravenously (i.v.; *n* = 6), intramuscularly (i.m.; *n* = 4–6), or subcutaneously (s.c.; *n* = 6) in anesthetized naïve pigs.

Respiratory frequency and body temperature were stable at normal values, and the fraction of inhaled oxide (FiO_2_) was 21% throughout the experiments (data not shown). The partial pressure of oxygen (pO_2_) decreased at 40 and 800 nmol/kg/min in the i.v. and s.c. experiments, respectively (Table 1).

Intranasal administration of PDNO caused small decrements of MPAP, dose-dependent lowering of MAP, and a distinct increase in FeNO at 2000 nmol (*n* = 6, Figure 4). Likewise, the preliminary observation of sublingual administration of PDNO showed a dose-dependent increase of FeNO and decrease of MPAP and MAP (*n* = 1, Figure 5).

**Figure 4.**
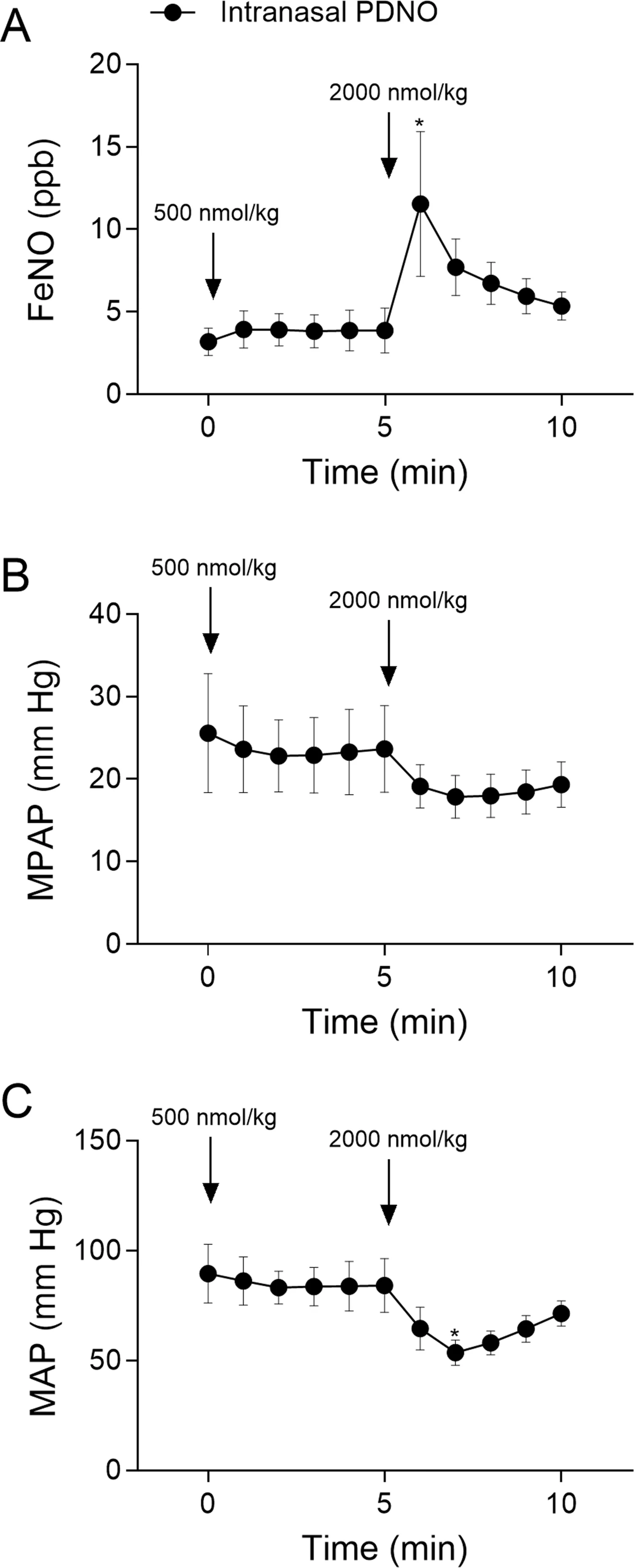
Effects of intranasal nitrosooxypropanol (PDNO) administration (*n* = 6) on fraction of exhaled nitric oxide (FeNO, panel A), mean pulmonary arterial pressure (MPAP, panel B), and mean arterial pressure (MAP, panel C) in anesthetized naïve pigs. Data are expressed as means with standard error of the mean. * represent statistical significance (*P* < 0.05) from baseline to maximal effect (1–2 min).

**Figure 5.**
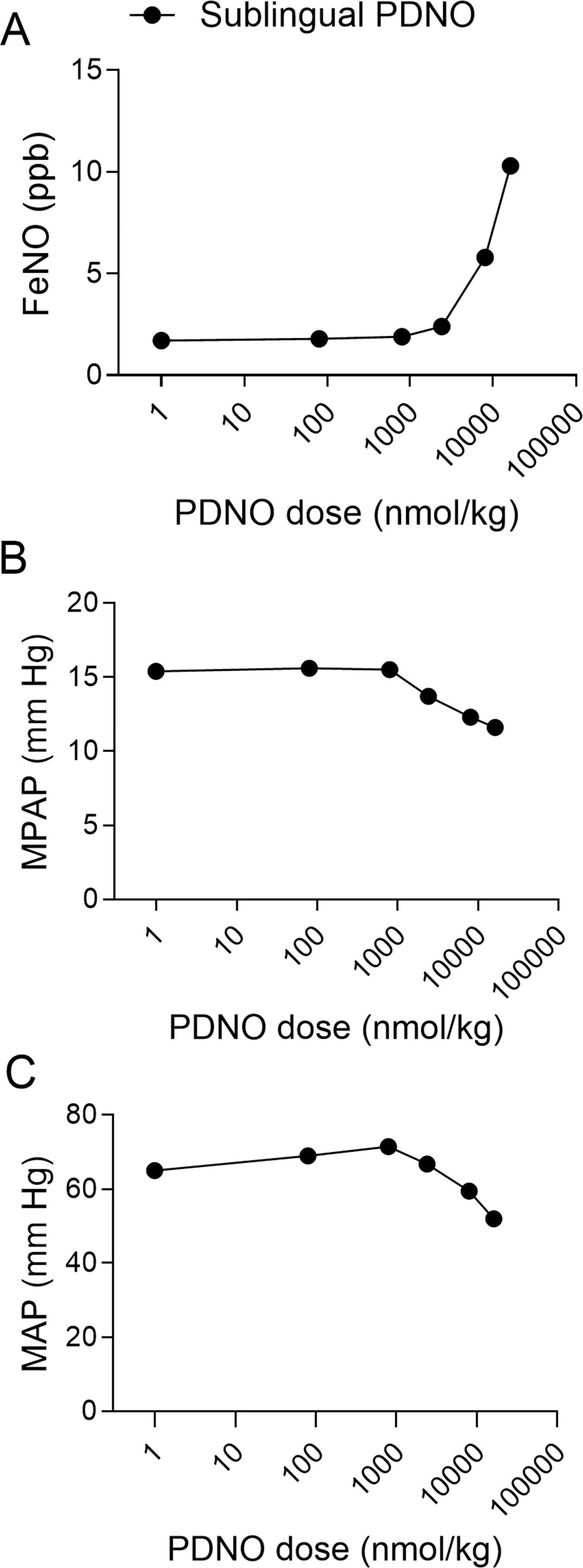
Effects of sublingual nitrosooxypropanol (PDNO) administration (*n* = 1) on fraction of exhaled nitric oxide (FeNO, panel A), mean pulmonary arterial pressure (MPAP, panel B), and mean arterial pressure (MAP, panel C) in an anesthetized naïve pig.

## Effects of PDNO in animals with elevated pulmonary pressure

During increased PVR, i.v. and s.c. infusions of PDNO led to dose-dependent decreases in MPAP and MAP (*n* = 6–7 Figure 6). FeNO also increased dose-dependently (Figure 6). The PVR and SVR were significantly decreased by the two highest doses of s.c. PDNO and PVR decreased at 15 nmol/kg/min by i.v. PDNO (Table 2). However, the ratio of PVR and SVR normalized during both i.v. and s.c. administration of PDNO, with a significant decrease from 15 nmol/kg/min, and 600 nmol/kg/min by i.v. and s.c., respectively (Figure 7).

**Table 2.** Hemodynamics and blood gases after infusion of nitrosooxypropanol (PDNO) in elevated pulmonary vascular resistance.

*U46619 + PDNO*			*HR* *(beats/min)*	*CO* *(L/min)*	*CVP* *(mm Hg)*	*PCWP* *(mm Hg)*	*SVR (mm Hg/L/min)*	*PVR* *(mm Hg/L/min)*	*pH*	*pO*_*2*_ *(kPa)*	*pCO*_*2*_ *(kPa)*	*MetHb* *(%)*
*I.v.*												
	U46619		113 ± 7	4.6 ± 0.5	10 ± 1	11 ± 1	18.0 ± 2.3	6.2 ± 0.9	7.58 ± 0.01	10.9 ± 0.4	4.5 ± 0.1	2.1 ± 0.2
	**5 nmol/kg/min**											
	9 min		112 ± 8	4.4 ± 0.4	10 ± 1		18.3 ± 2.1	5.9 ± 1.1				
	13 min		112 ± 8	4.3 ± 0.3	10 ± 0	11 ± 2	18.6 ± 1.9	5.9 ± 1.0	7.58 ± 0.01	11.0 ± 0.4	4.4 ± 0.1	1.8 ± 0.2
	**15 nmol/kg/min**											
	9 min		109 ± 9	4.3 ± 0.4	9 ± 0		17.6 ± 2.4	4.5 ± 1.0*				
	13 min		115 ± 9	4.2 ± 0.3	9 ± 0	11 ± 1	17.1 ± 2.5	4.5 ± 0.7	7.60 ± 0.01	11.6 ± 0.4	4.3 ± 0.1	2.2 ± 0.2
	**45 nmol/kg/min**											
	9 min		115 ± 10	4.4 ± 0.4	9 ± 0*		14.0 ± 1.7	2.9 ± 0.4*				
	13 min		114 ± 9	4.4 ± 0.3	9 ± 1	11 ± 0	14.1 ± 1.4	3.0 ± 0.3	7.60 ± 0.01	10.7 ± 0.6	4.3 ± 0.1	2.0 ± 0.1
*S.c.*												
	U46619		109 ± 8	4.1 ± 0.2	10 ± 1	9 ± 1	18.9 ± 1.3	5.8 ± 0.4	7.58 ± 0.01	11.2 ± 0.6	4.4 ± 0.1	1.8 ± 0.3
	**200 nmol/kg/min**											
	9 min		109 ± 7	4.7 ± 0.4	9 ± 1*		16.4 ± 2.2	4.5 ± 0.9				
	13 min		108 ± 7	4.8 ± 0.4	9 ± 1	11 ± 1	16.3 ± 2.2	4.5 ± 0.9	7.58 ± 0.01	11.6 ± 0.5	4.4 ± 0.1	1.5 ± 0.2
	**600 nmol/kg/min**											
	9 min		109 ± 7	4.8 ± 0.4	8 ± 0*		14.3 ± 2.3*	3.3 ± 0.7*				
	13 min		108 ± 7	4.7 ± 0.4	9 ± 0	10 ± 1	15.0 ± 2.1	3.6 ± 0.8	7.59 ± 0.01	11.6 ± 0.4	4.4 ± 0.1	2.0 ± 0.2
	**1800 nmol/kg/min**											
	9 min		111 ± 5	5.2 ± 0.5	8 ± 0*		11.8 ± 1.9*	2.4 ± 0.4*				
	13 min		110 ± 6	5.0 ± 0.4	8 ± 0	10 ± 1	13.0 ± 1.6	2.7 ± 0.3	7.60 ± 0.01	11.3 ± 0.5	4.3 ± 0.1	2.3 ± 0.2

^*^ Represents statistical significance (*P* < 0.05) from baseline to maximum effect (9 min after start of infusion).

Notes: Heart rate (HR), cardiac output (CO), central venous pressure (CVP), pulmonary capillary wedge pressure (PCWP), systemic vascular resistance (SVR), pulmonary vascular resistance (PVR), pH, partial pressure of oxygen (pO_2_), partial pressure of carbon dioxide (CO_2_), and methemoglobin levels (MetHb) during intravenous (i.v., 5, 15, and 45 nmol/kg/min for 15 min each, *n* = 4) and subcutaneous (s.c., 200, 600, and 1800 nmol/kg/min for 5 min each, *n* = 5) infusion of nitrosooxypropanol (PDNO) in anesthetized pigs with acute pulmonary hypertension (induced by a continuous i.v. infusion of the thromboxane A2 mimetic U46619). Data are expressed as means with standard errors of the means (SEM).

**Figure 6.**
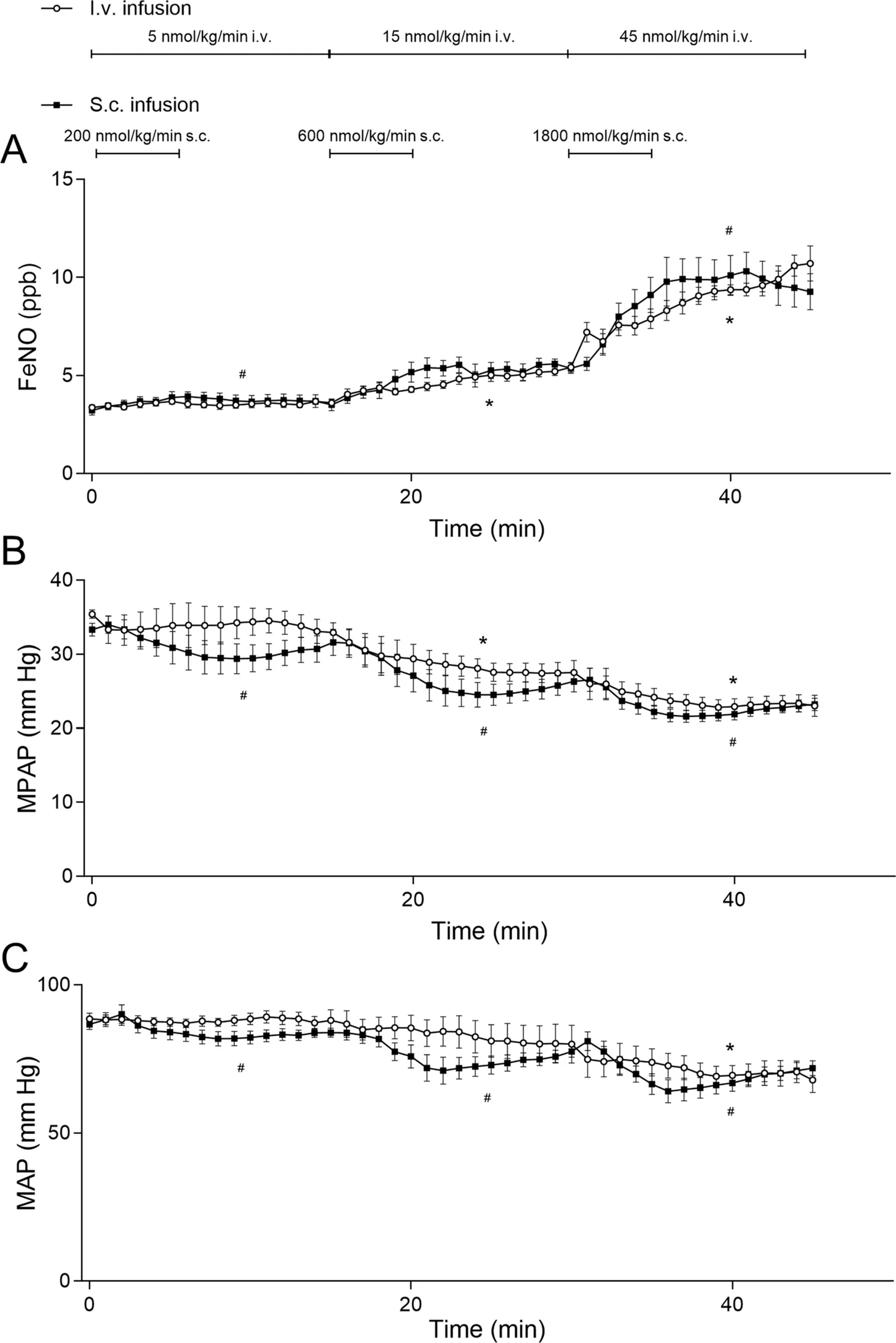
Effects of nitrosooxypropanol (PDNO) on fraction of exhaled nitric oxide (FeNO, panel A), mean pulmonary arterial pressure (MPAP, panel B), and mean arterial pressure (MAP, panel C) administered either intravenously (i.v.; 5, 15, and 45 nmol/kg/min for 15 min each, *n* = 6 or subcutaneously (s.c.; 200, 600, and 1800 nmol/kg/min for 5 min each, *n* = 7) in anesthetized pigs with acute pulmonary hypertension (induced by a continuous i.v. infusion of U46619). Data are expressed as means with standard error of the mean. * and ^#^ denote significant difference from baseline (*P* < 0.05) at maximum effect (9 min) for i.v. and s.c. administration, respectively.

**Figure 7.**
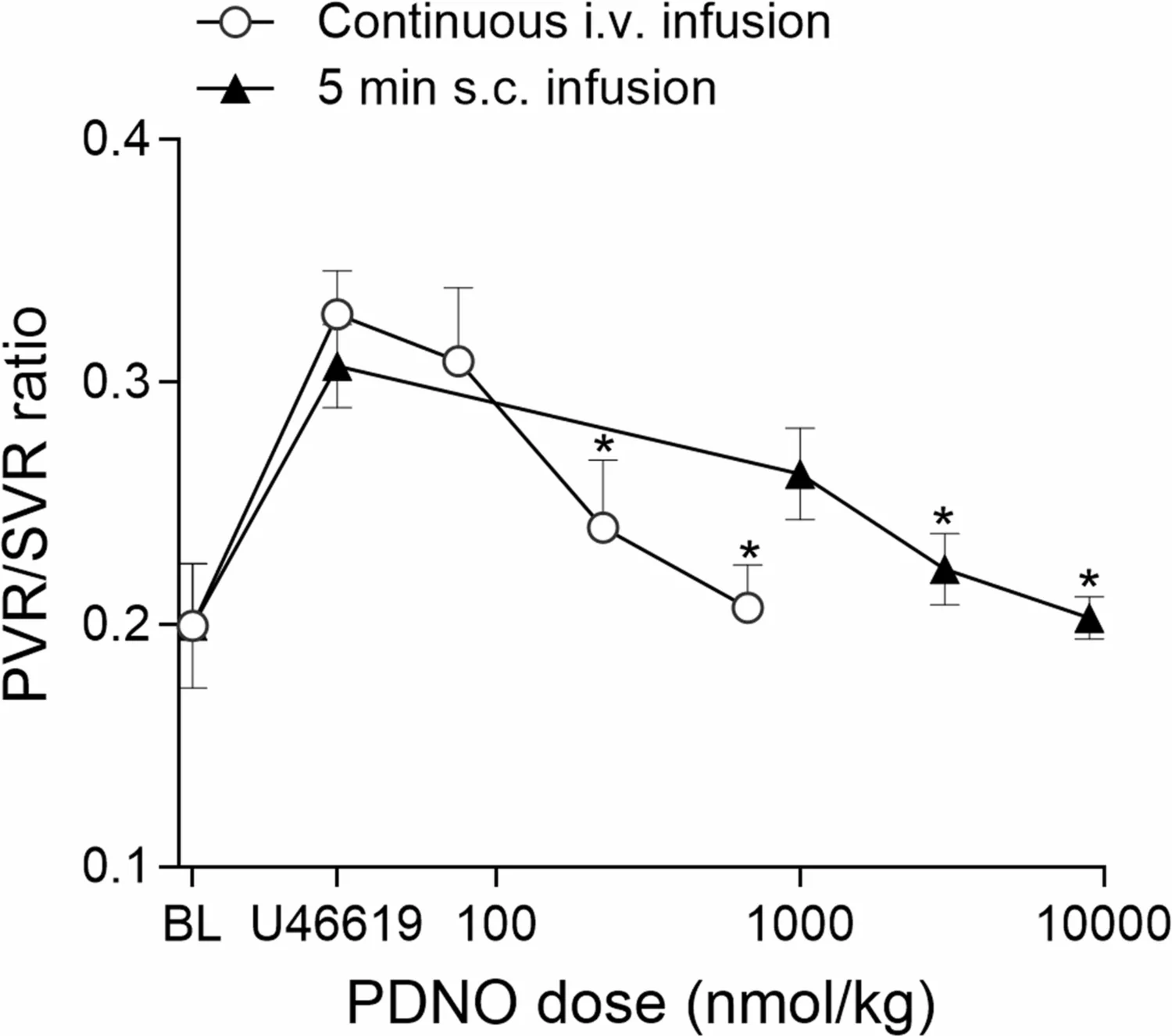
The ratio of pulmonary and systemic vascular resistance (PVR/SVR ratio) after intravenous (i.v.; 5, 15, and 45 nmol/kg/min for 15 min each, *n* = 6) and subcutaneous (s.c.; 200, 600, and 1800 nmol/kg/min for 5 min each, *n* = 7) treatment with nitrosooxypropanol (PDNO) in anesthetized pigs with acute pulmonary hypertension (induced by a continuous i.v. infusion of U46619). Data are expressed as means with standard error of the mean. * represents a significant difference from U46619 (*P* < 0.05) at maximum effect (9 min).

## Discussion

The current proof of concept study presents alternative routes for the administration of PDNO in a naïve and pulmonary hypertensive state in pigs. It suggests that PDNO exerts vasodilating effects by NO signaling when administered s.c., i.m., intranasally, and tentatively also sublingually. The exploratory study also suggests that PDNO may alleviate pharmacologically-induced pulmonary hypertension with selectivity for the pulmonary circulation, even when not administered directly into the bloodstream. To the best of our knowledge, no organic nitrite has previously been administered s.c., i.m. or intranasally and found to be bioactive. Neither has it been administered sublingually.

In this study, FeNO was used as a quantitative marker of NO generation from PDNO in recovery and half-time calculations. Endogenous pulmonary NO production, and thus FeNO, is modulated by multiple physiological and pathophysiological determinants (i.e. through obstruction of the pulmonary circulation, alterations in ventilation and respiratory gases, and inflammatory processes). In this study, however, all animals were mechanically ventilated with small variations throughout the experiments and animals were young and healthy. There was no change in baseline FeNO value throughout the experiments, indicating stable endogenous pulmonary NO production. When using exogenous NO sources such as NO donors, only a small fraction of NO given is excreted as exhaled NO. In the FeNO-based recovery and half-time calculations, a constant proportion of NO from the NO donor was assumed to be excreted to the exhaled gas. It is acknowledged that FeNO does not provide a direct quantification of total NO bioavailability (Agvald et al. 2006; Ragnoli et al. 2023). However, in controlled experimental conditions such as in this study, changes in FeNO during NO donor administration may be considered an indirect measure of bioavailability, as direct measurements of PDNO concentration in blood are not feasible due to a very short half-life in blood.

The FeNO-based recovery was lower when PDNO was administered outside the bloodstream, and also lower in s.c. administration compared with i.m. Given the very short blood half-life of PDNO observed in previous studies (Nilsson et al. 2011, 2017; Nilsson et al. 2018; Nilsson and Gustafsson 2019; Stene Hurtsen et al. 2020; Nilsson et al. 2020), the hemodynamic effects seen after administration in a peripheral compartment could be explained by slow-release of PDNO into the circulation, resulting in a delayed time to maximum hemodynamic effect (9 min) for s.c. and i.m. routes. The estimated FeNO-based half-life after s.c. and i.m. PDNO administration (20.5 and 6.2 min, respectively) could further support this notion. The pharmacokinetics of PDNO and other organic nitrites when administered via other routes than i.v. and inhalation have, however, not previously been studied. Given the limited vascularization of subcutaneous tissue, s.c. administration results in slow and sustained drug absorption. Critical determinants of absorption from the s.c. compartment include molecular size (larger molecules penetrate more slowly), solution viscosity (higher viscosity results in slower absorption), and local anatomical characteristics, such as vascularity and adipose tissue content at the injection site (McLennan et al. 2005; Kim and De Jesus 2025). In contrast, i.m. injections benefit from rich vascularity, allowing a more rapid systemic uptake, here demonstrated through a higher FeNO-based recovery and a shorter half-life of biological effects compared to s.c. administration. Key processes influencing absorption from the i.m. compartment include the diffusion of water into the depot, drug dissolution, movement through the delivery medium, and diffusion from the site of injection. Similar to s.c. administration, the proximity to blood vessels and local blood flow are essential factors influencing drug uptake (Zuidema JP and Duchateau 1998; Kim and De Jesus 2025; Gutierrez 2025). The intranasal route enables drug absorption via passive diffusion across the thin, highly vascular respiratory epithelium, allowing direct entry into the systemic circulation (Kim and De Jesus 2025). This explains the rapid biological response to intranasal administration of PDNO in this study. Similarly, drugs administered sublingually diffuse into the oral venous system and subsequently drain into the superior vena cava, thereby circumventing hepatic first-pass metabolism. For example, nitroglycerin undergoes over 90% first-pass hepatic metabolism and is, therefore, administered sublingually to ensure therapeutic efficacy (Kim and De Jesus 2025). Regarding organic nitrites, the literature has primarily focused on inhalational administration. Studies on alkyl nitrites, for example, describe highly lipophilic pharmacokinetic properties associated with readily partition into cellular membrane and adipose tissue, leading to rapid systemic absorption, prompt CNS distribution, and pronounced vasodilatory effects (Romanelli et al. 2004). The present formulation of PDNO is developed for i.v. use. The development of s.c. and i.m. formulations is challenged by highly variable tissue perfusion, unpredictable absorption kinetics, local tissue reactions, and physicochemical stability constraints, all of which can substantially affect bioavailability, depot formation, and local tolerability. These challenges may be particularly pronounced for lipophilic and chemically reactive compounds such as organic nitrites, due to their rapid partitioning into tissues, instability, and complex local biotransformation pathways (Miller and Megson 2007; Richter et al. 2012). Future studies are warranted to evaluate if formulations of PDNO for chronic use are manufacturable.

Available evidence further suggests that organic nitrites undergo less complex enzymatic bioactivation than organic nitrates. They may be directly reduced by hydrolyzation to NO, generate nitrosonium and other nitrosating species, react with thiols to form S-nitrosothiols (RSNOs), or undergo metabolism via hemoglobin- and xanthine oxidoreductase-dependent pathways. Biological signaling frequently occurs through these intermediates as well as through nitrite pools, HbNO complexes, and related intermediates, rather than exclusively via freely diffusing NO. Collectively, these processes are likely to influence compartmental distribution, biological half-life, and the spatial characteristics of local NO-mediated signaling (Feelisch and Noack 1987; Miller and Megson 2007). Whether PDNO itself or its metabolites are released from the depot formed following i.m. and s.c. administration cannot be determined from this pilot study. A potential direction for future research would be to quantify NO metabolites in plasma. Nonetheless, the present study indicates that the vasodilating effects of PDNO is via an NO species due to associations between increases in FeNO and other biological effects. Previous research offers support for the effectiveness of *in vivo* models for investigating the effects of NO donors utilizing measurements such as FeNO and systemic blood pressure (Persson et al. 1994; Cederqvist et al. 1994; Adding et al. 2005; Nilsson et al. 2011).

Concerns about excessive MetHb formation have been raised during the administration of organic nitrites (Abman and Kinsella 2002; Romanelli et al. 2004). Organic nitrites are non-polar, and hence, lipophilic, enabling passage into red blood cells (Nilsson et al. 2011). In this study, no elevated levels of MetHb were found, which is in agreement with previous studies where the substance was administered i.v. (Nilsson et al. 2011, 2017; Nilsson et al. 2018; Nilsson and Gustafsson 2019; Stene Hurtsen et al. 2020; Nilsson et al. 2020).

Organic nitrates are the most extensively studied NO donors. They are rapidly absorbed through mucous membranes, the gastrointestinal tract, and the skin. Thus, they are available in various preparations for delivery via several routes: oral tablets, buccal tablets, sublingual spray, transdermal ointment, and transdermal patch (Divakaran and Loscalzo 2017). They are also available in i.v. formulations and given as an intra-arterial bolus during angiography (Parker and Parker 1998; Islam et al. 2023). Nitroglycerin may exert effects on the pulmonary circulation, but treatment may provoke systemic hypotension in some patients and systemic hypoxemia in others (Packer et al. 1984). The half-life of nitroglycerin is approximately 1–4  min, and the biologically active metabolites have a half-life of approximately 40 min (Armstrong et al. 1982; Parker and Parker 1998). The mean transit time through a healthy pulmonary circulation is approximately 4–7 s, although longer in patients with PAH and congestive left heart failure (Neri et al. 1954; Slutsky et al. 1983; MacNee et al. 1989; Skrok et al. 2012; Swift et al. 2014; Cao et al. 2018; Houard et al. 2021), but this still limits the ability of GTN to produce a selective effect in the pulmonary circulation. Although GTN has been used to treat angina pectoris for over a hundred years, tachyphylaxis has complicated its chronic use (Parker and Parker 1998). Tolerance can develop within 12–24 h, reducing the clinical efficacy of organic nitrates. To mitigate this, specific extended-release formulas and dosing schedules are used, and a daily nitrate-free or low-exposure interval of 8–10 h is recommended (Parker 1993; Chrysant et al. 1993). Additionally, folic acid, L-arginine, vitamin C, and antioxidants have shown potential in preventing tolerance and endothelial dysfunction (Gori et al. 2001; Parker et al. 2002; Thomas et al. 2007). Nitrosooxypropanol has not, to date, been shown to induce tolerance development, nor does it appear to elicit cross-tolerance with nitroglycerin (Nilsson et al. 2018). Accordingly, treatment with an organic nitrite of this kind may offer a potential strategy to circumvent this clinical challenge, while still mediating the effects associated with NO activity.

The current study is a proof of concept; it is possible to administer organic nitrites through routes other than directly into the bloodstream and still achieve vasodilating effects with pulmonary selectivity in pulmonary hypertensive states. One potential application of an alternative administration route for an organic nitrite such as PDNO could be in the treatment of chronic PAH, a condition with few treatment options and a high mortality rate. Alternative administration routes may also have applications in patients in a prehospital environment, where i.v. access cannot be easily established, although this remains speculative. However, clinical feasibility in terms of dosing strategies, dosing reproducibility, route-specific pharmacokinetics, local tolerability, long-term safety, and regulatory considerations requires further evaluation and remains to be established before clinical translation.

### Limitations

The formulation of the organic nitrite PDNO used in this exploratory study was developed for i.v. use and may therefore have unknown side effects when administered via alternative routes. A formulation intended for specific administration routes may improve tissue bioavailability. Moreover, several administration routes were used in the same animal to reduce the number of animals according to the 3R (Russell and Burch 1959), potentially affecting the outcome since the pharmacokinetics is unknown and potentially bioactive substances may have remained in the animal after the first administration route. However, we used a washout period of a minimum of 1  h between administration routes, and the animals were hemodynamically stable and had baseline FeNO values before subsequent administration. We acknowledge that this approach may not completely exclude residual drug effects. The order of administration routes was not randomized to maximize the number of possible interventions per animal while minimizing the number of animals used according to the 3R. Blinding was not feasible due to the study design. Moreover, the number of animals used in each experiment was limited, affecting generalizability, particularly in the sublingual experiments, and it was not possible to perform a power calculation.

### Conclusion

This is a proof of concept showing novel administration pathways of organic nitrites. Nitrosooxypropanol exerts biological effects when administered subcutaneously and intramuscularly, with reasonable FeNO-based recovery. Furthermore, PDNO may also be administered intranasally with biological effects and tentatively even sublingually in this exploratory setting. Future work on formulations to enhance recovery and prolong delivery, and investigations into relevant disease models (for example, PAH models) and clinical settings, are warranted. Potentially, this development may contribute to an organic nitrite preparation for chronic use.

## Supplementary Material

Supplemental Material Author_Checklist_ARRIVE_updated.pdf

## Data Availability

All data supporting the findings of this study are available from the corresponding author upon reasonable request.
